# Direct interspecies electron transfer mechanisms of a biochar-amended anaerobic digestion: a review

**DOI:** 10.1186/s13068-023-02391-3

**Published:** 2023-10-03

**Authors:** Marvin T. Valentin, Gang Luo, Shicheng Zhang, Andrzej Białowiec

**Affiliations:** 1 Department of Applied Bioeconomy, Wrocław University of Environmental and Life Sciences, 51-630 Wroclaw, Poland; 2 Department of Science and Technology, Engineering and Industrial Research, National Research Council of the Philippines, Taguig, Philippines; 3 Benguet State University, Km. 5, La Trinidad, 2601 Benguet, Philippines; 4 Shanghai Key Laboratory of Atmospheric Particle Pollution and Prevention (LAP3), Department of Environmental Science and Engineering, Fudan University, Shanghai, 200433 China; 5 Shanghai Technical Service Platform for Pollution Control and Resource Utilization of Organic Wastes, Shanghai, 200438 China; 6 Shanghai Institute of Pollution Control and Ecological Security, Shanghai, 200092 China; 7 Department of Agricultural and Biosystems Engineering, Iowa State University, 605 Bissell Road, Ames, IA 50011 USA

**Keywords:** DIET, Biochar, Mechanisms, Biomass, Biogas, Anaerobic, Syntrophic

## Abstract

This paper explores the mechanisms of biochar that facilitate direct interspecies electron transfer (DIET) among syntrophic microorganisms leading to improved anaerobic digestion. Properties such as specific surface area (SSA), cation exchange capacity (CEC), presence of functional groups (FG), and electrical conductivity (EC) were found favorable for increased methane production, reduction of lag phase, and adsorption of inhibitors. It is revealed that these properties can be modified and are greatly affected by the synthesizing temperature, biomass types, and residence time. Additionally, suitable biochar concentration has to be observed since dosage beyond the optimal range can create inhibitions. High organic loading rate (OLR), pH shocks, quick accumulation and relatively low degradation of VFAs, and the presence of heavy metals and toxins are the major inhibitors identified. Summaries of microbial community analysis show fermentative bacteria and methanogens that are known to participate in DIET. These are *Methanosaeta*, *Methanobacterium*, *Methanospirillum*, and *Methanosarcina* for the archaeal community; whereas, *Firmicutes*, *Proteobacteria*, *Synergistetes*, *Spirochetes*, and *Bacteroidetes* are relatively for bacterial analyses. However, the number of defined cocultures promoting DIET is very limited, and there is still a large percentage of unknown bacteria that are believed to support DIET. Moreover, the instantaneous growth of participating microorganisms has to be validated throughout the process.

**Graphical abstract:**

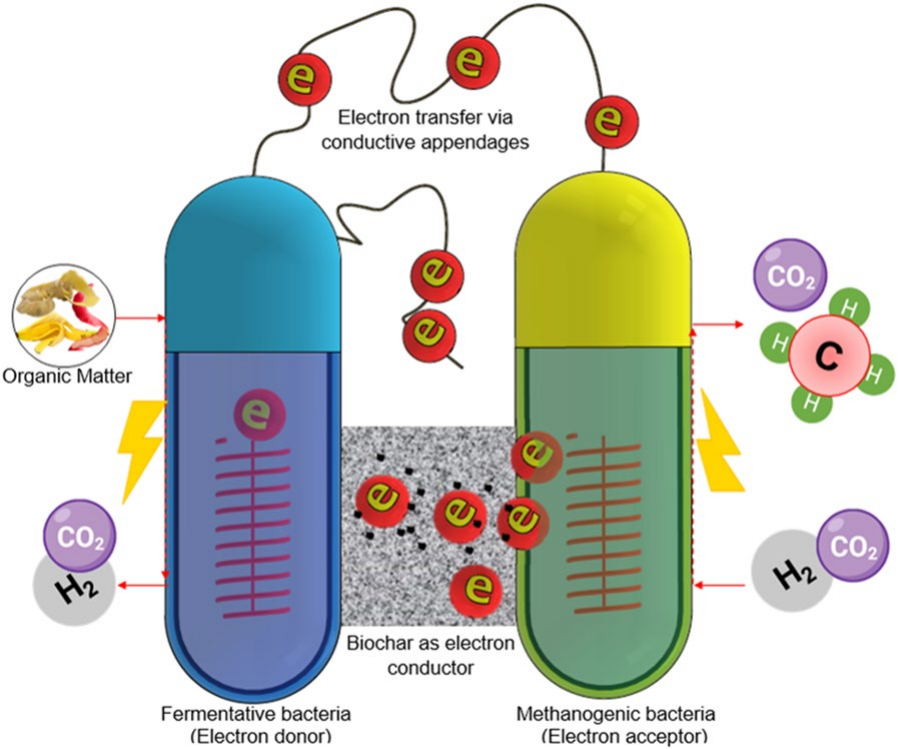

**Supplementary Information:**

The online version contains supplementary material available at 10.1186/s13068-023-02391-3.

## Fundamentals of anaerobic digestion

Anaerobic digestion (AD) is a favorable [1, 2], an economical [4], and an established efficient strategy to treat organic substrate while recovering energy and producing valuable fertilizer [5, 6]. AD also serves as a pollution mitigation measure [8]. It is by far the most feasible and pro-environment alternative waste valorization technology [9]. AD is carried out through the activity mediated by different guilds of microorganisms [8, 10, 11]. Likewise, AD is a process that involves a consortium of organisms that can effectively degrade complex substrates [10, 13]. These microorganisms are important to maintain, otherwise, it will result in inhibition [9, 14]. Ranges of biomass that can be treated include animal waste, food waste (FW) [15], agricultural waste [16], and an organic portion of municipal solid waste (MSW) [5, 6].

The degradation of biomass to biomethane involves hydrolysis, acidogenesis, acetogenesis, and methanogenesis carried out by corresponding distinct consortia of microbes [17–20]. In the hydrolysis stage, the organic substrates are converted into simple monomers such as lipids, proteins, and carbohydrates [22] through hydrolytic microbes like *Streptococcus* and *Enterobacter* [23]. Acidogenesis is an intermediate breakdown process between hydrolysis and acetogenesis that produces aldehydes, alcohols, and the predominant, important, and indispensable intermediate product which is the volatile fatty acids (VFAs) [23], such as the soluble monomers are degraded by acidogenic bacteria [22]. During acetogenesis, VFAs and other soluble monomers like long-chain fatty acids and sugars are converted into acetic acid, CO_2,_ and H_2_. Acetate can also be produced at this stage by the reduction of CO_2_ through the homoacetogenic bacteria and acetate to H_2_ and CO_2_ and vice versa via syntrophic acetate-oxidizing bacteria [22]. The concluding stage in the AD process is methanogenesis where H_2_, CO_2,_ and acetate are converted into CH_4_ by methanogens either hydrogenotrophic or aceticlastic. Throughout these processes, the performance of the AD of biowaste and biomass can be greatly affected by the inoculum-to-substrate ratio (ISR) [24] both in the lab-scale experiments and the full-scale performance of biogas plants.

The microbial community in an anaerobic digester is characterized by complex network of interactions, where each microorganism plays a specific role. The microbial community in an anaerobic digester is highly dynamic, and changes in environmental conditions can affect the composition and activity of the community. Understanding the microbial community in an anaerobic digester is essential for optimizing the process and improving the efficiency of organic waste treatment.

This work reports the mechanisms of biochar that stimulate DIET between syntrophic microorganisms and subsequent influence on methane production; lag time improvement; production and degradation of VFAs; and enrichment of microbial community in defined cocultures with their responses to BC supplementation.

## Challenges in an AD system

Despite rigorous research works, AD is restricted by several challenges like low methane production; instability [8, 25, 26]; methane quality due to complexity in the physical and chemical properties of substrates [28, 29]; quality assurance of the digestate [29]; the need to conduct additional process such as size reduction [30, 31] to hasten degradation [29, 33]; complexity in balancing fermentative and methanogenic microorganism [7]; and low organic loading rate (OLR) (3.5 gVS l^−1^ d^−1^) [34] are yet to be solved. Furthermore, small variations in the AD process can have an adverse effect, especially at the sensitive stage [19, 35]. Common instability in AD is caused by high OLR [8, 36–38], pH shock, and other inhibition effects brought by the accumulation of VFAs [40], NH_3_ [41], and even those that are initially present in the feedstock such as heavy metals (HM) [42]. Kutlar et al. [35] noted that the conversion of VFA to methane by methanogens is relatively slow. Accumulation of toxic inhibitors also causes instability [5]. This toxin can be from the substrates themselves which will disturb the syntrophic functions of the bacteria [43] and even retard microbial growth [8, 44] that can lead to instability as manifested by decreased pH, rapid VFAs accumulation [6], and low CH_4_ production [8, 36, 37]. The slow growth rate of methanogens can cause a longer hydraulic retention time (HRT) [5, 45].

To attain stability in the AD system, there should be a healthy environment for the microorganisms to survive for them to fulfill their respective functions [16, 46]. This is particularly because methanogenesis is the most sensitive stage in the AD process [19, 21, 47] such that even a small deviation of operating conditions from the threshold level can result in inhibition [48]. Additionally, since substrates for AD are rich in nitrogen (N) and carbon (C), the system is prone to excessive accumulation of organic ammonia (NH_3_) and ammonium (NH_4_^+^) produced during protein breakdown [49] and volatile fatty acids (VFAs) which are considered major inhibitors to methanogens [50]. VFA and NH_3_, at a safe level, can serve as essential nutrients to support the growth of microorganisms. Some literature reported ammonium levels to be safe at 1200 mg l^−1^ [51] or even at a range of 1700–1800 mg l^−1^ [49, 52]. Ammonia inhibition is common in the nitrogenous substrate [49, 52, 53], animal manure, and slaughter by-products [53, 54]. It is then noteworthy to emphasize the appropriate microbial community for efficient anaerobic digestion at minimal or reduced inhibition effects [56]. Syntrophic relations of bacteria are defined by their ability to transfer electrons at a stable and fast rate [57] to survive considering optimum parameters are met such as pH [58]; organic loading rate (OLR) [59]; and temperature [60] among others. Their survival and growth are well proven to promote better AD processes as indicated by improved CH_4_ production and lag time. Interestingly, direct interspecies electron transfer (DIET) was found as a new pathway for electron transfer between bacteria and archaea, facilitated by carbon materials such as biochar (BC) as electron shuttles and was claimed to be more efficient than another mode like the interspecies electron transfer (IET) facilitated through H_2_ [61]. The quality of AD performance is affected by the syntrophic bacteria and archaea [43] and the efficiency of electron transfer [6, 62]. Thus, a syntrophic environment allowing fast acclimatization of microbial growth through DIET for subsequent faster methane production has to be further explored [11].

### Volatile fatty acids

VFA, though an important component in the anaerobic food chain towards methane production [19, 21], has been reported to be the major cause of process failure in AD when they accumulate [21, 63, 64] and can also generate malodor. VFA mainly comprise propionic acid, butyric acid, and acetic acid [65] and their accumulation is accompanied by subsequent H_2_ partial pressure build-up [66]. The partial pressure must be kept low in order for the VFA to be degraded in the anaerobic digestion [66]. Recent works suggest that BC can effectively accelerate the degradation of VFA [23, 26, 67]. Specifically, Kaur et al. [67], recorded a decrease of propionic acid to 1.46 g l^−1^ of AD added with wheat straw BC at 10 g l^−1^ concentration. Li et al. (2021) reported a 68.9% decrease in butyrate coupled with an increase in CH_4_ production [68]. Li et al. [65] investigated the concentration of BC (1, 5, and 10 g l^−1^) co-digestion with corn straw and sewage sludge and compared the change in VFA from the maximum concentration against the end of digestion. VFA, from the experiment, tends to have a higher percent decrease at 5 g l^−1^ suggesting that VFA, particularly acetic and propionic acids, accumulation and degradation are affected by the optimum dosage of BC [65].

Accumulation of VFA is also a result of high OLR [35, 69] and bacterial disruption [70]. Such inhibition can be suppressed through the addition of biochar [71, 72]. Xu et al. [73] observed better performance in CMs amended reactors, compared to the control, even at a high OLR of 12.0 kg COD. (m^3^ day)^−1^. This is coherent with the observation of Dang et al. [74] that CH_4_ production was possible and was even improved even under high OLR with the supplementation of carbon materials. Meanwhile, VFAs level, which serves as a reliable marker of instability in AD [75], can undergo a transformation into CH_4_ through two pathways: the conversion of acetate to CH_4_ by acetoclastic methanogens or the syntrophic acetate-oxidizing bacteria (SAOB), and the transfer of electrons from VFA to CO_2_, resulting the production of CH_4_ [35]. The transfer of electrons from VFA to CO_2_ is performed by hydrogenotrophic methanogens owing to the process being hydrogen-mediated interspecies electron transfer (HMIET) [35, 57]. The electron can also be transferred via formate as the mediator and the process is called interspecies formate transfer (IFT) [76] DIET is another pathway alternative to HMIET and employs the use of conductive pili of fermentative bacteria to transfer electrons from the oxidation of VFAs to the methanogens [77]. This process can be enhanced by conductive materials such as biochar [78].

### Heavy metals

Several studies have proven that BC can effectively reduce or absorb pollutants in AD like heavy metals, toxins, or antibiotics [4, 79]. Zhao et al. [26] provided a detailed review of the application of biochar to reduce hazardous compounds such as heavy metals. In this report, it is noted that BC efficiency to mitigate toxins is associated with the increase in CH_4_ production, VFAs degradation, and improvement of lag time [26]. A meaningful suggestion can be drawn from the report that BC sorption efficiency can be further studied in terms of the number of heavy metals that were removed by the BC either those that adhered to the surface or loosely and tightly bounded ones. Zhang et al. [4] reported that higher immobilization of HMs (Cd, Ni, Cu, Cr, and Zn) in an AD of sewage sludge was due to the increased number of active sites and FGs of the supplementing biochar, MnFe2O_4_-BC. In addition, a higher pyrolysis temperature at 700 °C was favorable in minimizing Cu and Zn [80].

### Total ammonia nitrogen (TAN)

Total ammonia nitrogen comes in the form of free ammonia nitrogen (FAN), NH3, and its ionized form NH4^+^ is another inhibitor in AD [8, 52, 81]. This was first documented by Hansen et al. [82]. FAN is necessary for VFA and CH_4_ production [83]. However, FAN at an excessive level (1500 mg kg^−1^) will inhibit methanogens leading to the accumulation of VFA [52]. Yenigün et al. [49] concluded that FAN is more toxic than TAN as it caused a 50% reduction in methane production at a concentration ranging from 0.0017 to 1400 mg l^−1^ [8]. NH_3_ can be controlled with the addition of BC [84]. The threshold value of TAN at 1700–1800 mg l^−1^ has been identified to critically affect AD operation that causes process inhibition when exceeded [26, 49]. A TAN level of 150 to 1200 mg l^−1^ can have toxic effects on anaerobes [49]. Rajagopal et al. [52] added that methanogens will be suppressed at TAN levels above 3000 mg l^−1^. The addition of BC is reported to regulate the rise of TAN [65] and even increase AD tolerance at high TAN concentrations as proven by improved AD performances [81, 85–87]. Li et al. [65] observed that TAN concentration was effectively alleviated, with BC addition at 444.79 mg l^−1^ compared to the control with a TAN value of 1016.45 mg l^−1^. Khalil et al. [89] observed that rice straw BC was effective (43%) in adsorbing as high as 4.5 mg g^−1^ ammonium from an aqueous solution. Sarkhot et al. [92] confirmed that BC is an effective material to adsorb ammonium as high as 5.3 mg g^−1^ from dairy manure effluent [89]. Similarly, Poirier et al. [81] reported that CCM supplemented reactor had higher ammonia tolerance as manifested by a 25% improvement in the lag phase even if the TAN concentration was 1900 mg l^−1^. Yu et al. [85] noted a significant improvement of over 96% CH_4_ production at an AD stressed at 6000 mgl^−1^ TAN. In addition, Zhai et al. [93] concluded that higher SSA resulted in a significant reduction of ammonia. Zhao et al. [26] observed that particle size significantly affects ammonia mitigation. These two qualities are important not only in ammonia adsorption, but also in other important operations in AD such as facilitating microbial immobilization as discussed in the earlier section [94]. Lü et al. [50] confirmed that ammonia alleviation was improved at larger particle sizes such that immediate NH_3_ alleviation was observed at BC of size 2–5 mm coupled with improved CH_4_ production and lag phase compared to BC with the particle size of 0.5–1.0 mm and 75–150 μm that took longer time to response.

## Properties of biochar

Biochar is an electrically conductive and stable carbon-rich material synthesized through the thermal degradation of organic materials in an oxygen-starved reactor at high temperatures [95, 95] ranging from 180 to 1500 °C [98, 98]. It has been widely studied owing to its characteristics to promote and enhance methanogenic reactions in the AD system [101, 102]. Properties of biochar include porosity, surface area (SA), electrical conductivity (EC) [103], high cation exchange capacity (CEC) [3, 104], and FGs present at the surface [3, 105]. Additional properties are pore size, specific surface area (SSA), and elemental compositions [15]. Among these properties, porosity has more weight on AD performance [15]. The SA of BC [106] (130m^2^ g^−1^) has a significant role to host microbial colonies [102] and increase interaction with the environment [107]. BC supplies ample surface area for microbial attachment and promotes biofilm formation, [108] which can reduce the lag time (41–45%), enhance VFAs degradation, and increase the CH_4_ production rate (23.0–41.6%) [102]. BC yield is affected by biomass type, pyrolysis temperature, and heating rate [109]. The pyrolysis temperature influences the chemical composition (CC) of biochar such as P, Ca, and Mg being increased with temperature while C and N were inverse with temperature due to combustion and volatilization [110]. H and O can be reduced at increased temperatures, resulting in the development of positive properties of biochar such as polarity [111], pH, and aromatization [96, 112, 113]. SSA and pore volume also increase with temperature [13, 114]. For instance, rice straw biochar pyrolyzed at 500 °C has a respective SA and pore volume of 34.4 m^2^ g^−1^ and 0.028 cm^3^ g^−1^ [115] while BC from rapeseed plant synthesized at the same temperature has 15.7 m^2^ g^−1^ and 1.150 cm^3^ g^−1^ [116]. Biomass sources can also affect other aspects of BC such as in terms of yield [26], and porosity which is usually higher in plant-based material [117]. Lignocellulosic biomass has usually a higher BC yield [118] than other sources like animal manure [119] which is usually of higher ash content [120].

## Properties of biochar influencing DIET

Biochar exhibits FGs [105, 121] capable of supporting microbial growth [122] which is necessary to facilitate electron transfer [19]. Several studies have fully established that BC can stimulate DIET in the AD system resulting in shorter lag time which is often credited to its conductive properties [5, 123] in addition to its ability to support microbial growth [6]. BC is an efficient electron shuttle and both its EC and redox-active moieties (RAMs) are important in the electron transfer between bacterial cells [124]. Quinones and phenazines are RAMs that facilitate and stimulate electron transfer [125]. Yu et al. [124] observed the presence of quinone moieties on biochar that were synthesized at higher temperatures and these are important in bacterial IET. The addition of BC facilitates the formation and degradation of VFAs [126]. Sunyoto et al. [102] investigated the influence of the concentrations of BC on simulated carbohydrate food waste as substrate was added and found that cultures with BC added degraded VFAs faster than without BC during the first 14 days. Shanmugam et al. [6] found that variability in ECs of BC is affected by the natural ash composition in addition to biomass types and pyrolysis temperatures. Kalderis et al. [127] affirmed that EC increases with formation temperature. This is also coherent with the observation of other authors [128, 128–131].

EC is a major BC parameter that affects the electron transfer between bacterial cells [6, 72, 124]. Kato et al. [134] observed that methanogenesis rate and lag time were highly improved by conductive property. In addition, Li et al. [135] observed that DIET did not occur in insulated carbon materials suggesting that DIET was stimulated by the conductivity of the additives.

Redox-active moieties are another important property of biochar, derived from the FGs, that allows efficient electron transfer [6] and are not mainly due to EC and SA. This now explains why BC, even though it has significantly low EC (2.1–4.4 μS cm^−1^) compared to GAC (3000 μS cm^−1^), can better enhance methanization and improves the lag phase [35]. This is strengthened by the findings of Wang et al. [136] that BCs of lower ECs exhibit more redox-active organic FGs that improved the CH_4_ production rate.

Measures that were implemented to address the identified AD limitations in “Challenges in an AD system” section were subjecting the biomass to preliminary processing like size reduction; modification of AD reactors [28, 33, 137]; application of additives [27, 52, 138]; use of high substrate-to-inoculum ratio (SIR) for quicker stabilization period; and use of additives to immobilize microorganisms [37]. Consequently, most additives increase the operating cost of the AD system [37, 139]. Biochar was found to have comparative performance with other additives at a relatively low and reasonable cost [37] in addition to its widespread application due to the presence of favorable physical and chemical qualities [29]. Overall, the addition of biochar, compared to a non-supplemented AD reactor, has been reported in the literature to improve AD by facilitating biofilm formation and mitigating inhibition [102, 140, 141] as manifested by improved performance parameters presented in Table 1.

**Table 1:** Selected performance parameters of anaerobic digestion of biomass supplemented with biochar with their corresponding improvements reckoned from control

Conductive materials	Substrate	Favorable change concerning control			References
		CH4 yield (%)	Lag phase reduction (%)	COD removal (%)	
BC	Acetate	22.6	1.5	–	[142]
	Ethanol	14.4	7.1	–	[142]
	Kitchen waste	30	–	7	[143]
	Food waste	33.2		60–88	[144]
	Simulated carbohydrate-rich food waste	6.2	41	–	[102]
	Sewage sludge from WWTP	55.9	61	–	[4]
	Glucose	–	–	21.6	[126]
Hydrochar	Artificial N-rich substrates	32		27.1	[53]
Pyrochar	Artificial N-rich substrates	–	–	10.8–20.3	[53]
GAC	Kitchen waste	26	29		[145]
	Dog food	Increased by 16-fold	–	212	[146]
	Fat, oil and grease, and waste-activated sludge	6.7–13.4	200–400	55.1–58.5	[147]
PAC	Dry anaerobic digestion of sewage sludge	49	16.6–58.3	–	[148]
	*Flammulina velutipes* residues	–	26.6	–	[149]

Furthermore, BC enhances stability [3, 44] by adsorbing major inhibiting compounds and elements like NH_3_, HM, and toxins [29, 150]. The presence of rich FGs, aromatic groups, and amine makes the BC effective to adsorb toxins [27] while at the same time hastening the degradation of VFAs [37, 151]. Besides, the porous structure of BC offers space for microorganisms to thrive and make colonies [37, 100, 152] and can also hold nutrients on its large surface area (SA) to support microorganisms [150]. BC is a good electron conductor and can accelerate electron transfer between fermentative bacteria and methanogens [5, 35, 44], compared with other materials, which is highly important in enhancing anaerobic methane production [121]. Optimum BC dosage is also important to consider as it can reduce CH_4_ production and even worsen the lag phase when overdosed or underdosed [4, 65]. Li et al. [65] noted a remarkable decrease in the lag phase at BC dosage of 5 g l^−1^ and consequently, dosage at 10 g l^−1^ and 1 g l^−1^ showed a decline in methane production rate. Dudek et al. [141] observed that maximum biogas production of Brewer’s spent grain (BSG) added with BC at higher concentrations (20–25%) decreased from 85.1 to 61.0 dm^3^ g^−1^ dom (dry organic matter). On the other hand, there were some studies claiming that BC-amended reactors had not shown methane increase such as wood chips biochar as reported by Yuan et al. [153]. This is attributed to a lower concentration of quinone and hydroquinone in wood BC that resulted in reduced electron transfer capability [153].

Supplementation of AD with BC increases tolerance to inhibition and at the same time promotes DIET [65]. This was proven by the increase in CH_4_ production and 25% reduction in lag phase in an AD with a stress level of 1900 mg l^−1^ total ammonia nitrogen (TAN) level which is beyond the threshold [81] as reported elsewhere [49, 51]. Similarly, Lü et al. [50] confirmed that methanization was accelerated when added with BC even under double risk inhibition of ammonia and acid.

In terms of economic advantage and applicability, BC has widespread environmental applications such as contaminants-removing agents in wastewater (WW) [154], soil amendment [155], and carbon sequestration [156] making it economically superior over other common conductive materials like activated carbon (AC) since it can be generated from biowaste [39], and even from municipal solid waste [157]. AC, on the other hand, though it has superior quality especially in terms of electrical conductivity (EC) than BC [158], its production cost is 10 times higher than BC [126] and it needs to be recovered from the digestate for further use to reduce cost [158]. Residual BC can be used as fertilizer with immediate benefit to improving soil fertility [37, 126, 156, 158]. Besides BC production through established technology like pyrolysis entails a cheaper cost [29] as it requires low heat [63] compared to AC and zeolite and it is formed from agricultural residues [14] that are usually cheap or even free [27]. Besides, biochar treatment through pyrolysis as reported by Syguła et al. [158] is safer than other modes of thermal conversion. Moreover, biochar properties can be manipulated depending on the application by varying preparation parameters like temperature, residence time, and types of biomass [121]. From the environmental aspect, biochar can contribute substantial environmental benefits in the reduction of carbon emissions [159]. BC can also be applied to plants without further modification which indicates widespread application [78].

## Direct interspecies electron transfer

DIET is now considered a modern pathway of electron transfer [57] in improving CH_4_ production [35, 160]. It facilitates the reduction of organic compounds [161] like VFAs, alcohols, C_2_H_6_O to acetate, and H_2_/CO_2_ through syntrophic microorganisms [158, 162]. DIET promotes better syntropy between acetogens and methanogens leading to improved AD resistance against inhibitions [163] and promoting efficient biological conversion [164]. A balanced syntrophic relationship of these bacteria speeds up biomass oxidation and reduction of CO_2_ to CH_4_ [165, 166]. DIET was first documented by Summers et al., (2010) in an experiment of *Geobacter metallireducens* cocultured with *Geobacter sulfurreducens* which illustrated favorable aggregate formation in mutants that are incapable of interspecies hydrogen transfer (IHT) suggesting cooperative partners among the bacteria [77]. This was attested by Lovley et al. [167] to be more advantageous since the need to produce hydrogen to shuttle electrons is discarded and the energy in producing H_2_ can be saved by the syntrophic partners. DIET is stimulated through different syntrophic biological partners categorized as biological (bDIET) such as microorganisms that possess conductive appendages such as G. *metallireducens* or c-type cytochrome [76] while DIET initiated by conductive materials such as carbon materials is categorized as conductive mineral mediated (mDIET) [19, 168]. Several kinds of nonbiological materials which have been previously studied to enhance DIET [72] were BC [40, 69, 72, 103, 126, 144, 153, 169–178]; activated carbon (AC) [173, 180]; granular activated carbon (GAC) [73, 74, 136, 142, 145, 147, 173, 181–187]; powdered activated carbon (PAC) [142, 148, 149]; graphite [147, 172, 184]; and graphene [189, 190] among others. Wang et al. [76] found BC as the second most used CM accounting for around 20.9%, next to GAC (24.3%).

The role of electron transfer conductor is important to promote the syntrophic growth of coculture [186]. Summers et al. [77] and Rotaru et al. [191] observed that coculture did not grow when gene pilA was deliberately deleted in the case of *Geobacter metallireducens* and *Geobacter sulfurreducens*. This highlights the importance of conductive pili to promote DIET [168]. However, coculture metabolism can still be possible even if the conductive pili is deleted through the supplementation of biological electrical connections or conductive materials [192]. Chen et al. [123] found out that biochar in a cocultured with *G. metallireducens* and *G. sulfurreducens* or *M. barkeri* with C_2_H_6_O as electron donor was able to stimulate DIET and with the phenomenally close contact of the cells with the biochar suggesting that biochar is capable of serving as a conduit for electron and that PilA deficient *G. sulfurreducens* even outperformed the cocultures of wild-type strains of both bacteria in terms of converting fumarate to succinate. Similar to the observation of Kato et al. [134] that electron flow between syntrophic partners is possible through a nonbiological conductor that manifested increased CH_4_ production and reduced lag time.

### Cell attachment

In an AD not supplemented with carbon materials, syntrophic microorganisms *G. metallireducens,* and *G. sulfurreducens* formed aggregates for electron transfer with the rich presence of c-type cytochrome [77]; whereas, microorganisms such as *G. metallireducens* and *M. barkeri* were observed to tightly associate with conductive materials but not form aggregates as compared to an environment without carbon materials where microorganisms form aggregates to create electron shuttles through the cell-to-cell connection [57]. Aggregation of cells is usually observed when the only mode of electron transfer is via biological connections [185]. This suggests that electron transfer aside from biological connection can be made possible using conductive material as an electron shuttle [57] through DIET as discussed earlier [57, 77, 193]. Lee et al. [62] observed that exoelectrogens and hydrogenotrophic methanogens were enriched on the surface of conductive materials suggesting that DIET is evident in nonbiological conductors.

### Conductive appendage

Another evidence for the occurrence of DIET is the intentional deletion of conductive pilin which inhibits CH_4_ production under AD conditions where the only electron transfer is using the biological connection [192]. Despite pilin deficiency, the syntrophic microorganisms can transfer electrons with the presence of conductive material amended AD. Chen et al. [192] performed an experiment initiated with pilin-deficient *G. sulfurreducens* in which CH_4_ production is the same as that of cultures initiated with wild-type strains, suggesting that carbon material was able to serve as an electron shuttle that facilitated DIET between microorganisms.

### C-type cytochrome

C-type cytochrome OmcS, just like conductive pili, is important for biological extracellular electron connection [77] and responsible for promoting DIET [185]. Previous works investigated cocultures of strain initiated by c-type cytochrome, OmcS deficient with the amendment of carbon materials were still be a be to metabolize. For instance, OmcS-deficient *G. sulfurreducens* was reported by Chen et al. [192] to metabolize ethanol (C_2_H_6_O) for the production of succinate. This encompasses the observation of Liu et al. [185] that OmcS deletion still proceeded to the metabolism of C_2_H_6_O in the addition of carbon material.

## DIET-related microorganisms

**Fig. 1:**
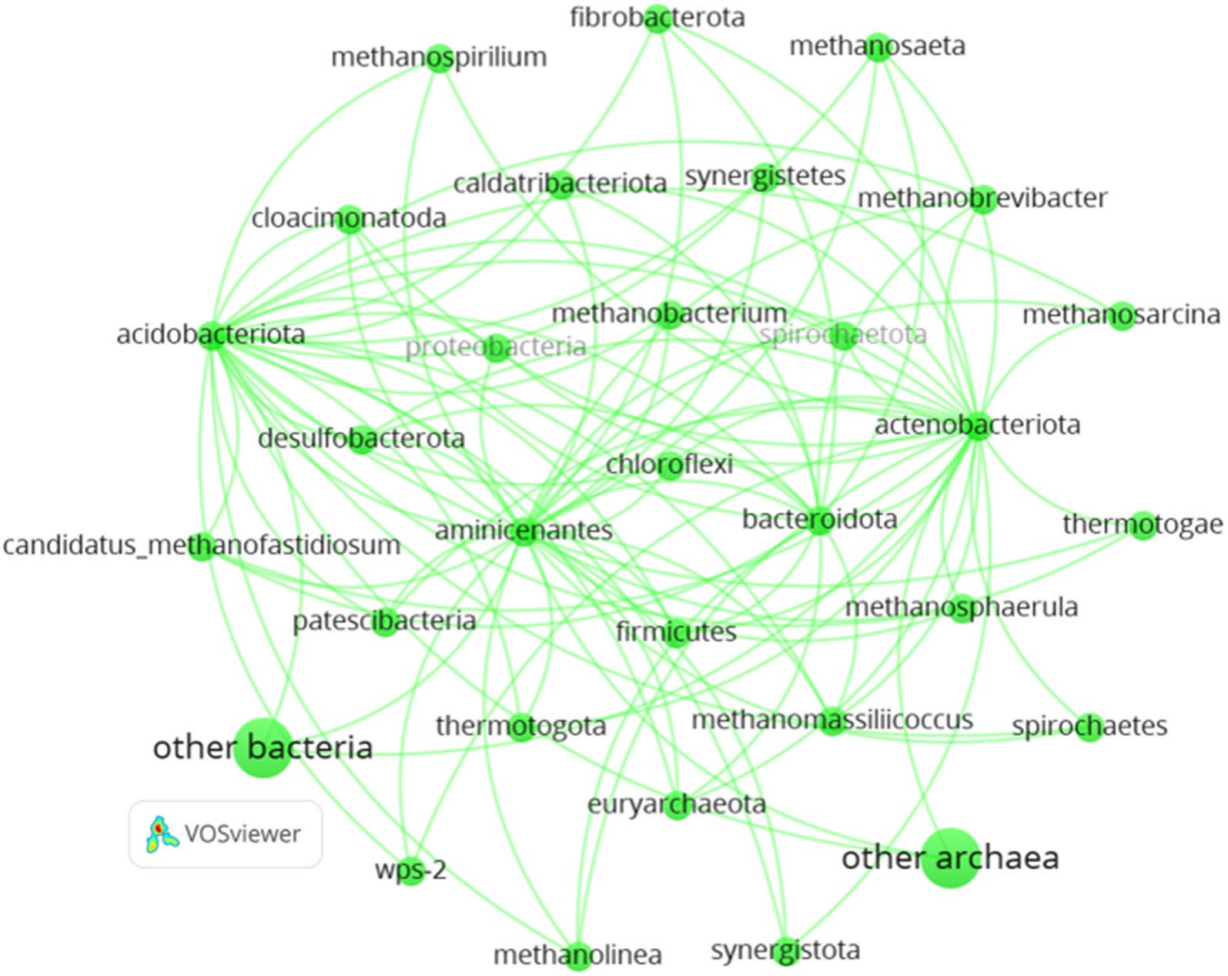
Network map of the archaeal and bacterial community that is relatively abundant in AD supplemented by biochar prepared using VOS viewer software (Additional file 1). The size of the circle indicates the relative abundance of the methanogens while lines represent the co-occurrence among the community

The occurrence of DIET in an AD reactor is usually expressed in terms of the microbial community known to participate in DIET and their subsequent enrichment during the AD process [35]. Kutlar et al. [35] mentioned that DIET is carried out between the syntrophic bacteria (acetogens) and archaea (methanogens). These relatively abundant acetogens and methanogens are shown in Fig. 1. The microorganisms are represented by the circles connected by lines. These are the relatively abundant microorganisms co-occurring in anaerobic digestion. The lines indicate co-occurring among the microorganisms indicating that a certain microorganism is likely to co-exist with other microorganisms with which it is linked. However, there is only a little information about the diversity of methanogens promoting DIET [186]. Few studies were conducted relative to the population of microorganisms in defined cocultures and their performance throughout the AD process, like in the study of Lu et al. [50] where the growth of known microorganisms was monitored from the inoculum to the early stage up to the completion in the digestion of glucose amended with BC. Additionally, most works reported that the community for both bacterial and archaeal analysis comprised a relatively higher percentage of unknown microorganisms, suggesting that more studies should be conducted considering these details. To better understand how the microorganisms participate in DIET, it is presented in this section the previous research works that have studied BC amendment with the effects on the DIET-related microorganisms [186, 192, 193] (Table 2).

**Table 2:** Compendium of experimental observations manifesting DIET between cocultures of defined microorganisms where one serves as an electron donor and the other as an electron acceptor in an AD system

Coculture		Culture medium	Manifestations of DIET	Refs.
e-donor	e-acceptor			
*G. metallireducens*	*G. sulfurreducens*	• Ethanol and fumarate • With BC	• Ethanol was metabolized and fumarate was reduced to succinate on day 2 • Cells were attached to BC but did not aggregate	[123]
		• Without BC	• Ethanol metabolism started at day 30	
		• Ethanol and fumarate • With carbon cloth	• There was a syntrophic metabolism of ethanol and a reduction of fumarate to succinate on day 2 • Higher metabolism when the carbon cloth was doubled • Acetate did not accumulate • Cells were dispersed	[192]
		• With cotton cloth	• No ethanol oxidation and succinate production due to the very low conductivity of the carbon cloth	[192]
*G. metallireducens*	None	• With BC	• Ethanol metabolized slowly with an increase in acetate • BC served as an electron acceptor	[123]
		• Without BC	• No ethanol metabolism	
*M. barkeri*	None	• Pure culture	• Not ethanol metabolism	
*G. metallireducens*	*M. barkeri*	• Ethanol	• Ethanol was converted to methane • Transient accumulation of acetate • Microorganisms were attached to BC but did not aggregate • BC served as an electrical conductor between the two species and not through cell-to-cell electron transfer	[123]
		• Without BC	• Not ethanol metabolism	
		• Ethanol as the sole electron donor	• Ethanol metabolized to methane on day 7 • Transient accumulation of acetate • Formation of intertwined aggregates (100–200 μm) that shared electrons via DIET • *M. Barkeri* was able to participate in DIET	[186]
		• Pure culture	• No metabolism of ethanol and no acetate formed	[186]
		• No GAC	• Ethanol started to metabolize after 39 days	[186]
*G. metallireducens* wild-type	*G. sulfurreducens* is incapable of producing pili	• Carbon cloth	• The succinate produced is comparable to the coculture initiated with wild-type strains • Cells were tightly attached to carbon cloth at day 10 of incubation • This indicates that the removal of pili did not inhibit the attachment of cells	[192]
*G. metallireducens* is incapable of producing pili	*G. sulfurreducens* wild-type	• Carbon cloth		
*G. metallireducens* wild-type	*G. sulfurreducens* Omcs deficient	• Carbon cloth	• There was succinate production	[192]
*G. sulfurreducens*	None	• Ethanol & fumarate • With carbon	• No ethanol metabolism or fumarate reduction even with carbon cloth	[192]
*G. metallireducens*	None			
*Desulfovibrio vulgaris*	*G. sulfurreducens*	• Ethanol • With carbon cloth	• The cloth did not accelerate metabolism	[192]
*G. metallireducens*	*M. barkeri* strain	• Ethanol	• Cocultures without cloth required metabolized ethanol at day 40 • Cocultures with carbon cloth started to metabolize ethanol began at day 10 • Cells were not closely associated with each other	[192]
pilA-deficient or Gmet 18668 gene deficient strain *G. Metallireducnes*	*M. Barkeri*	• No GAC	• Did not metabolize ethanol and no methane was produced	[186]
		• With GAC	• The amendment of GAC in the coculture allowed the pili-deficient strain G. Metallireducens to transfer an electron to M. Barkeri resulting in the production of methane • Proof that GAC can serve as a substitute for pili to shuttle electrons	
*P. carbinolicus*	*M. barkeri*	• Ethanol	• There was growth in the coculture • A steady accumulation of acetate was observed • No multispecies aggregates formed illustrating that DIET requires cell-to-cell for electron transfer • *M. Barkeri*, using H_2_, metabolized a little of the acetate produced by *P. Carbinolicus* • *M. Barkeri* is the first methanogen known to use both H_2_ and or electrons from DIET to reduce CO_2_	[186]
*P. carbinolicus*	*G. sulfurreducens*	–	• No aggregate formed, suggesting that close physical contact was not necessary for interspecies H_2_ transfer	[186]

### Archaeal community

Doping of BC enriches methanogens especially those identified to participate in DIET and most of these were *Methanosaeta*, *Methanobrevibacter*, *Methanobacterium*, *Methanomassiliicoccus*, *Methanosarcina*, *Methanospirillum*,* and Methanolinia* [65, 94, 121]. Luo et al., (2015) observed, in the digestion of glucose supplemented with BC, that *Methanobacterium* was the most enriched methanogen species followed by *Methanosaeta* and *Methanosarcina* constituting 90% of the total community [126]. *Methanosaeta* and *Methanosarcina* were proven to conduct DIET [35] and their enrichment is an accepted indication of electron transfer via DIET [186]. Coherent to the observation of Li et al. [40] where *Methanosaeta* was most abundant, followed by *Methanospirillum*, *Methanobacterium,* and then *Methanosarcina* in the digestion of FW supplemented with BC. These methanogens, being the major bacteria responsible for methane production, are dominating in the mesophilic condition in addition to *Methanococcus* Spp., *Methanobrevibact*er Spp. [19, 194, 195].

The addition of BC, because of its large specific surface area (SSA), enriched the genus *Clostridium* which shortened the fermentation period in the AD system [7]. Wang et al. [39] observed that *Methanosaeta* and *Methanosarcina* were slightly inhibited at high organic loading shock but they are relatively abundant comprising around 62.08% and 10.66% of the archaeal population in the BC-amended reactor as compared to the control with the relative abundance of 29.12% and 3.34%, respectively. *Methanobrevibacter* was observed by Li et al. [143] to account for around 61% of the archaeal community from sludge methanogenic digester whereas it accounted for 3.2% in the BC reactor. On the other hand, there are unknown species constituting a large percentage of the overall microbial population [40, 48, 50, 65, 126, 196]. The addition of BC can also increase the detectability in the community which was illustrated in the experiment of Wang et al. [39] where the other unidentified microorganisms constitute more than 50% of the relative abundance of the taxonomic classification observed in non BC reactor but were reduced in BC-amended reactor.

### Bacterial community

The influence of biochar supplementation in AD can be further explained by the composition of enriched bacteria. The most enriched group of bacteria were *Firmicutes*, *Bacteroidota*, *Proteobacteria*, and *Actinobacteriota* were relatively abundant as measured toward the end of the AD process and constitute around 52% of the total taxonomic bacterial community at the phylum level [7, 65, 68, 69, 93, 94, 148, 161, 182]. Pan et al. [7] reported similar observation of relatively abundant bacteria from AD amended with mushroom biochar pyrolyzed at 550 °C in addition to *Synergistetes*, *Acidobacteria*, and *Euryarchaeota* with *Proteobacteria Firmicutes* being enriched. Wang et al. [39] reported a slight decrease (2%) of *Syntrophomonas* at high organic loading shock even amended with BC but *Geobacter* was mostly enriched to 22.6 fold higher than the control reactor.

### Microbial enrichment

**Fig. 2:**
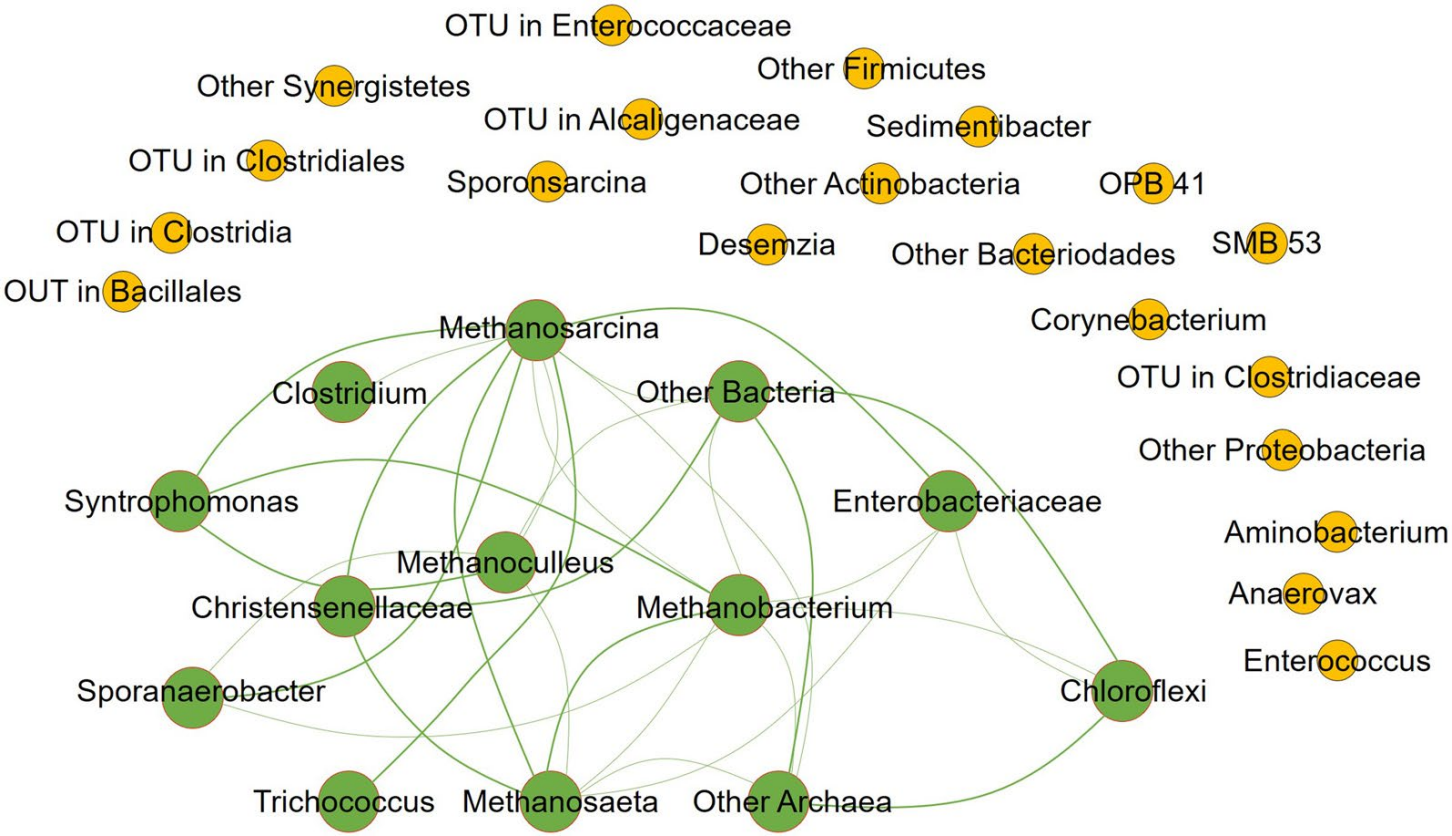
Acetogens and methanogens enriched after biochar supplementation in the anaerobic digestion process

The progressive growth of bacteria in AD could be substantial information to monitor how a particular microorganism behaves throughout the process either in terms of growth, resistance to inhibition, and recovery rate when suppressed. Lü et al. [50] revealed from their work on the AD of glucose with BC subjected under ammonium stress levels of 0.26, 3.5, and 7 g N l^−1^ of which the bacterial and archaeal communities were observed in the inoculum, early stage, and during the final stage of AD. Likewise, Li et al. [65] monitored the changes in the microbial population of both bacteria and archaea during the maximum CH_4_ production stage and at the end of the digestion. With this, from among the identified microbes and anaerobes, some were able to grow throughout the process which is believed to exhibit syntrophic relation, however, others were suppressed indicating they were not compatible with the microbial community (Fig. 2). In the figure, the circle indicates the various microorganisms. At the end of the AD process, the microorganisms that were suppressed were construed not to co-occur with the microbial community. Co-occurring microorganisms that show an increase in their population are linked together by the lines. However, those that were suppressed are not connected with lines and are outside the co-occurring microorganisms.

Works on AD supplemented with BC synthesized at different temperatures and biomass types report a variety of information as to how the AD system was affected. Overall, the summary indicates an improvement in AD (Table 3).

**Table 3:** Summary of previous research works on BC-amended reactors and the subsequent influence of BC on AD performance and the enhanced microbial population

BC	Treatments	AD Performance	Enhanced microbial population	Ref
Fruitwoods (800 °C)	BC levels (2, 4, 6, and 8 g/l); and BC sizes (2–5 mm, 0.5–1 mm, and 75–150 μm)	• CH_4_ decreased at increased glucose due to the increase in soluble microbial products at higher stress levels • Smaller particles are better in VFA degradation	• *Methanobacterium* was most enriched followed by *Methanosaeta* and *Methanosarcina* • *Syntrophomonas* increased to 29.2% followed by *Clostridiaceae*	[126]
Fruitwoods (800–900 °C)	Total ammonium concentration of 3.5 g-N/L	• *λ* = 12.7%; R_max_ = 10.1%; VFA = 66 mmol-C l^−1^	• Inoculum was highly populated by *Proteobacteria* and Firmicutes • *Enterobacteriaceae* was detected at the end of AD • *Clostridium* and *Porphyromonadaceae* were enriched at the later stage • *Methanobacterium* increased from 30%(inoculum) to 92.1% (early stage) to 65.6% (final stage) • *Methanosaeta* and *Methanosarcina* were suppressed	[50]
Fruitwoods (800–900 °C)	Total ammonium concentration of 7.0 g-N/L	• *λ* = 23.8%; Rmax = 23.5%; VFAs = 66 mmol-C l^−1^	• *Enterobacteriaceae* was detected at the early stage and was enriched to the final stage • *Methanogens* and *Methanosaeta* were suppressed	[50]
Sawdust BC (650 °C) at 20 min retention time with fractional size 3.5–25.9 μm	BC addition ratios of 8.3, 16.6, 25.1, and 33.3 g-l^−1^	• The CH_4_ yields for 25.1 and 33.3 g-l^−1^ BC were lower than the control (1070.0) • CH4 decreased at an increased dosage of BC	• The highest CH_4_ yield (1136.6 ml-l^−1^) was observed at 8.3 g-l^−1^ • Lower methane production was recorded from 33.3 g-l^−1^ • Higher BC dosage had more propionic acid accumulated which led to low pH • The addition of BC promoted biofilm formation	[102]
Canola meal (700 and 900 °C) and; switchgrass (500 °C); and Ashe juniper (400 and 600 °C)	Biomass types and temperature	• Effective reduction in lag time • CH_4_ improvement was 72% for SBC-500, and ABC-400 (71%), compared to GAC (40%) and PAC (24%) • The COD reduction was 94% for GAC, whereas 93% for PAC, 94% for SBC-500, 93% for ABC-400, and GLU was 81%	• *Lewinella* was the most enriched archaea (18%, SBC-500), (16%, ABC-400), (19%, GAC), (18%, PAC), and (16%, Glucose) followed by *Bacteroides*, *Bacillu*s, and *Dechloromonas* • Methanogens like *Methanothermobacter* were most enriched at 76.2, 60.1, 64.1, 74.5, and 77.7% for ABC-400, SBC-500, GAC, PAC, and glucose, respectively, followed by *Methanosarcina*	[6]

## Inhibitor-resistant AD

**Fig. 3:**
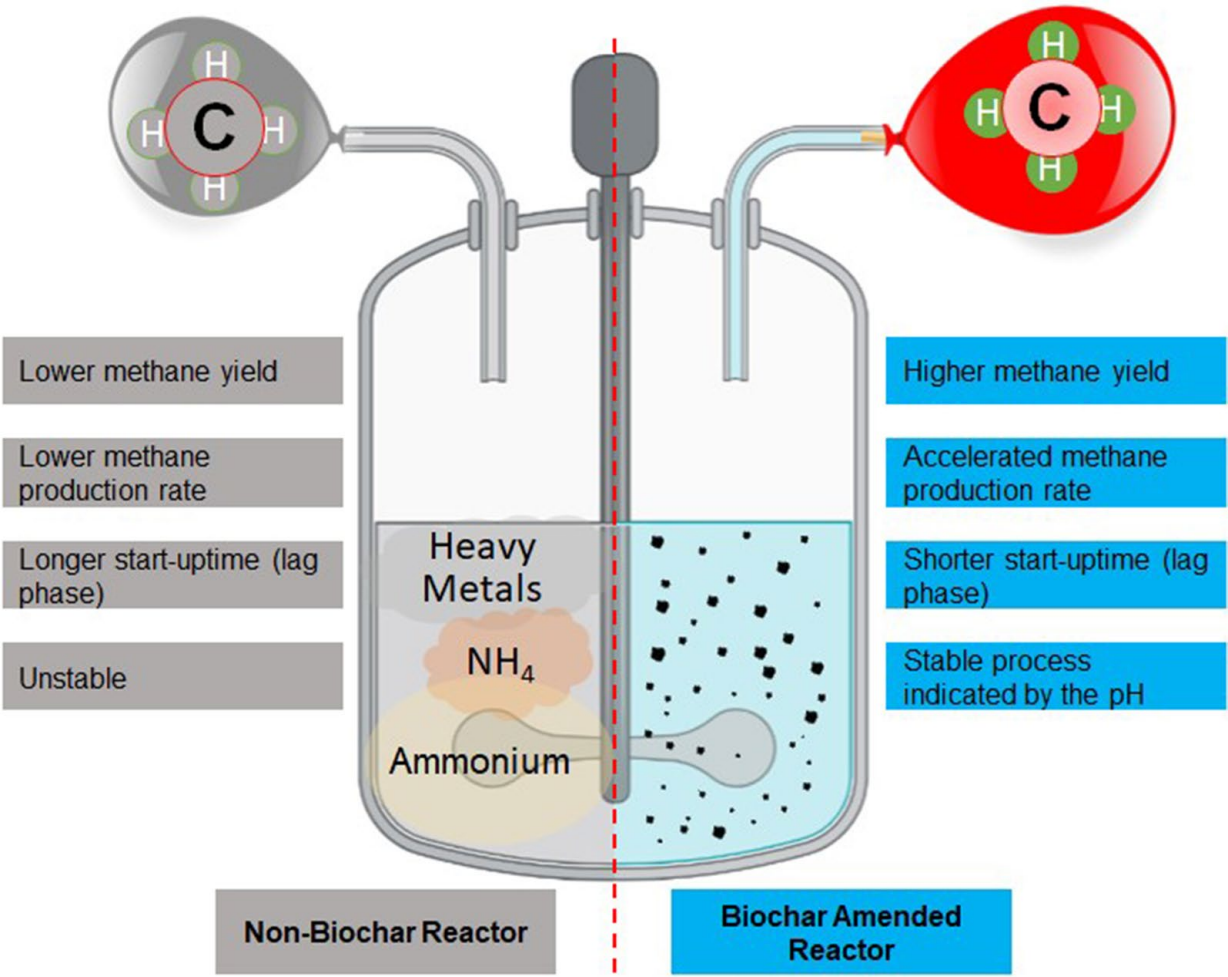
Comparison of anaerobic digester following DIET metabolic pathway against anaerobic reactor following non-DIET pathway

Strong resistance to inhibitors will result in more efficient electron transfer among syntrophic microorganisms [197]. The ideal AD environment offers a well-balanced population between fermentative and methanogenic bacteria resulting in optimal accumulation and timely degradation of intermediates such as VFA, NH_3_, and NH_4_^+^ for the production of methane as manifested by a measurable indicator in the AD system like an increase in CH_4_ yield, production rate, and decreased lag time (Fig. 3). This AD environment has been well researched to be the promising influence of DIET which is facilitated by conductive materials like BC. On the contrary, methanogenic production following a non-DIET-based pathway is characterized as an imbalanced AD system as a result of the excessive accumulation and relatively slow degradation of those intermediates formerly mentioned. Development and accumulation of extreme inhibitors become more dominant in this kind of reactor. In terms of process efficiency, economics, and quality of AD products, DIET intervention has to be embraced.

## Summary of previous reviews

This section presents a compendium of related recent review works with selected objectives and the corresponding significant findings and conclusions relative to the addition of BC in an AD environment (Table 4).

**Table 4:** Previously conducted research reviews on the use of biochar as an additive toward an enhanced AD performance with emphasis on the roles of biochar in promoting DIET

Objectives related to biochar and DIET	Significant findings	Year	References
Compare AD amended with different CCMs, concerning CCMs type, particle size, dosage, electrical conductivity (EC), redox properties, and AD operational factors, such as temperature and organic loading rate (OLR)	• BC is the second most used CCM next to GAC • Literature reported that CCM addition enhanced CH_4_ production • Some BCs like wood chips caused inhibition of CH_4_ production which is related to its lower quinone and hydroquinone content • BC with high electron donating capacities is important • VFA accumulation due to high OLR resulted in an imbalance in microorganisms, pH drops, and process failure was mitigated by CCMs • During hydrogen build-up causing VFA accumulation and pH drop, CCM can alternatively accept electrons during the process to proceed with VFA degradation • NH_3_, a necessary nutrient for microorganisms but at high concentration (1700–1800 mg l^−1^) can cause inhibition but methanation and lag phase will still be improved with CCM amendment • Other inhibitors like sulfate have been classified to react in a way that its corresponding reducing bacteria will compete with methanogens over OM resulting in reduced CH_4_ but this can be mitigated through CCM • BC is rich in FG giving redox properties that allow electron transfer other than EC (2.1–4.4 μS cm^−1^) explaining why BC is more efficient to promote DIET even though its EC is extremely low compared to GAC (3000 μS cm^−1^)	2022	[35]
Properties and functionality of BC and their role in AD and economic challenges	• Properties of BC are affected by the pyrolysis environment such as temperature and retention time • Temperature and biomass types determine the formation of FGs (carboxyl, hydroxyl, phenolic hydroxyl, carbonyl groups) of BC • Microbial activity and relative abundance are greatly affected by the properties of BC and this can be done by meeting the optimum nutrient requirement in the AD • BC is a multi-function agent such as an inhibitor adsorbent and serves as an environment for microbial colonization, electron conductor, and pH buffer • NH_3_ or NH_4_^+^ ions (FAN) released at high concentrations in the AD inhibit methanogens that will cause the VFA to accumulate. TAN should then be pinned to a safe level (1500 mg/kg) and this can be more economically and easily mitigated using BC addition as compared to pH and temperature adjustment, maintaining C:N and pre-treatment • VFA accumulation and sudden pH drop are caused by unbalanced acidogenic and methanogenic microorganisms and VFAs accumulation can be controlled with the introduction of *Syntrophomonas* spp. and *Syntrophobacter* spp.	2021	[27]
Investigate the production process of BC and its physicochemical characteristics; and identify the mechanism of BC that improves AD	• Feedstock types and the synthesizing parameters are major factors that influence the yield and characteristics of BC • Synthesizing environment and the feedstocks types affect the electron transfer capability of BC • The ability of biochar to shuttle electrons is positively influenced by increased temperature • VFA inhibition is coupled with the higher H_2_ partial pressure • Electro-active microorganisms are enriched with the BC addition and the VFA metabolism shifts from IHT to DIET • BC with larger size (2–5 mm) quickly alleviate NH_3_ inhibition and led to a high CH_4_ production at reduced time, followed by medium-sized (0.5–1 mm) particles, while the slower response in smaller size biochar (75–150 μm)	2020	[26]

## Conclusions

The mechanisms of electron transfer in an AD via DIET as facilitated by the addition of BC were reported in this paper and the following observations were drawn:

1. The capability of BC to promote DIET is affected by its major physical and chemical properties which include particle size, presence of FGs, electrical conductivity, and redox-active moieties. These properties are significantly affected by the pyrolysis temperature, followed by residence time and biomass types.
2. The FGs in BC are important for the degradation of VFAs and the adsorption of toxins and heavy metals in addition to their porous structure.
3. The presence of redox-active moieties in BC allows the improvement of methanization even though its EC is a 1000-fold lower than other carbon materials.
4. The metabolism of OM in the AD system is carried out between syntrophic archaea and bacteria by donating and accepting electrons from each other. BC has been reported to serve as a shuttle for electron transfer in place of biological electrical shuttles like conductive pili and OmcS c-type cytochrome.
5. Biochar is capable of hosting microbial growth on its surface (loosely bound), between micropores (tightly bound), or even in the supernatants. These partitions of biochar are unique to specific types of microorganisms. The reason why some bacteria are not detected at the start of the AD process but emerged after some time was because they were tightly bound inside the biochar.
6. The dosage of biochar is related to the capacity of the AD system to absorb heavy metals, sulfate, TAN and FAN, and VFAs oxidation. The situation where VFAs become a major inhibitor is when it accumulates quickly with very slow degradation by the methanogenic bacteria as influenced by OLR and HRT. The biochar served as a temporary substrate for microbial growth.

## Recommendations

The following recommendations to further improve DIET activity in an AD system as manifested by enhanced CH_4_ production and lag phase are drawn:

1. It is highly recommended that AD be supplemented with BC of smaller particles like 0.5-1 mm or 75–150 μm since it was documented that at such a range of size, CH_4_ production was better than the larger particles like 2.0 to 5.0 mm [126].
2. Pyrolysis temperature is a crucial factor that influences the major properties of BC like FGs, CEC, EC, and even SA have been investigated in several works. Considering the economic aspect of the BC production lower temperature may be used so long as it will not compromise the optimum values of BC properties.
3. FGs in BC such as carbonyl, hydroxyl, and phenolic hydroxyl as reported by to be affected by temperature. These are major factors in adsorbing contaminants and counteracting inhibitors, but they can diminish when the pyrolysis temperature treatment is exceeded or not met. With this, it is recommended that BC may be produced at a temperature ranging from 400 to 500 °C and the optimal temperature must be carefully investigated.
4. BC’s capability, aside from its physical and chemical properties, to either adsorb or absorb certain adsorbates is also affected by the types of contaminants present or being developed in the AD. In principle, the adsorption begins at the surface of the BC by attachment and then eventually forms denser and tight aggregation on BC surfaces. In addition, adsorbates find their way inside the BC through the pores until saturation. At this time, the BC will no longer adsorb and absorb contaminants. With this, it is important to consider the proper proportion of biochar to the possible quantity of contaminant in the AD. From this, it is necessary to characterize the types of contaminants in a particular substrate and their growth. This information can lead to the appropriate timing as to when BC can be added to the reactor. It is then possible to add BC at a specified time during the operation and not at once during the start of the AD process.
5. The efficient flow of electrons largely defines the success of biomass conversion to CH_4_ which is claimed to be facilitated by BC between the acidogenic bacteria and methanogens under the DIET pathway. With this investigation of the instantaneous flow of electrons from a defined group of bacteria to archaea and to emphasize the rate at which the biochar can conduct electrons could be prospect research.
6. While several microorganisms can participate in DIET, most studies dealt with the enriched population at the end of the study. It would be more objective to consider how these microorganisms grow throughout the process beginning from the AD operation to establish their growth rate. Likewise, most studies have presented PCR results and scanning methods that a large percentage of the bacterial and archaeal population is still unknown. These unknown microbes could be contributing to the DIET reaction and knowledge about them is important to further understand the function of biochar in the microbial community.
7. BC SSA and porosity may be further modified to optimize their capability to serve as thriving objects for syntrophic microorganisms.
8. Bacterial and archaeal population progressive growth could be an important aspect to further investigate. This is to establish the instantaneous change in the quantity of a particular microorganism and how is it related to other response variables in the AD.
9. Ammonia inhibitions were mitigated by biochar, but not in higher concentrations (3.1–6.6 g TAN kg^−1^). The detailed interaction between biochar and microorganisms relating to ammonia oxidation must be studied.
10. A mechanism to evaluate a direct and visual flow of electrons between syntrophic microorganisms has to be established to further validate DIET and not only based on AD’s overall performance.

### Supplementary Information

**Additional file 1**: **Figure S1**. Data file preparation in CSV format containing the microorganisms that were subjected to VOSviewer network map creation. **Figure S2**. Sample VOSviewer network map of microorganisms.

## Data Availability

All data are given in the manuscript.
