# Supplementary material for: Direct interspecies electron transfer mechanisms of a biochar-amended anaerobic digestion: a review
Source: Biotechnol Biofuels Bioprod. 2023 Oct 3;16:146. doi: 10.1186/s13068-023-02391-3 (PMC10546780; doi:10.1186/s13068-023-02391-3)
Supplement: Supplementary file 1 — Additional file 1: Figure S1. Data file preparation in CSV format containing the microorganisms that were subjected to VOSviewer network map creation. Figure S2. Sample VOSviewer network map of microorganisms. [file 13068_2023_2391_MOESM1_ESM.docx]

**Additional materials**

This document shows how Figures 1 and 2 were created in the manuscript. Recent research has identified several bacteria and archaea that were relatively abundant in the anaerobic digester after adding biochar. The procedure is outlined as follows which may help understand the clarity of the figure.

1. **Creation of *.txt file**. The identified bacteria and archaea per study were prepared in a *.txt file format as shown in the figure below. The bacteria and archaea that are relatively abundant were grouped per study(source) and are separated by semicolon. The space between Study_1 and the group of microorganisms is separated by a tab. The file was saved in *.txt format.


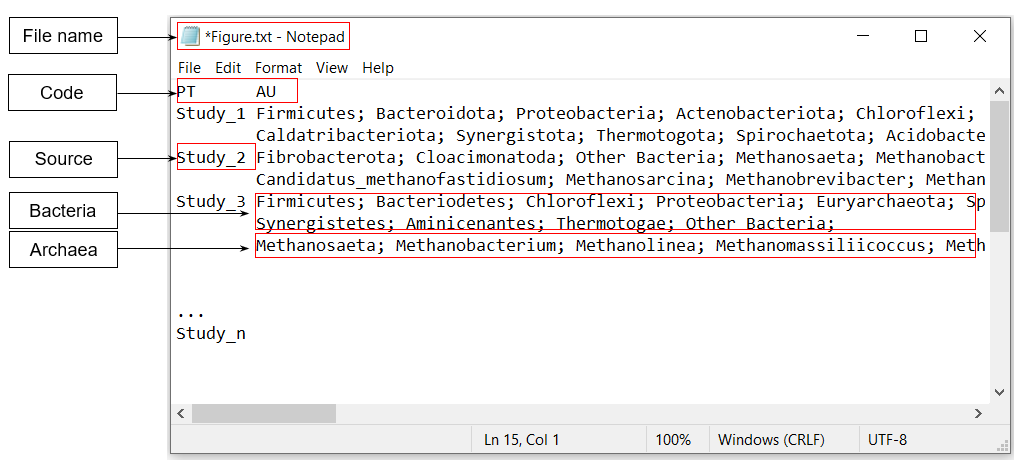


Figure S1. Data file preparation in CSV format containing the microorganisms that were subjected to VOSviewer network map creation.

1. The file can now be fed to the VOS viewer. It can be done as follows:

- Open the VOSviever;
- Click on “create”;
- Choose “Create a map based on bibliographic data”;
- Click next;
- Choose “read data from bibliographic files”;
- Click next;
- Open the desired file;
- Click next and then proceed to the creation of the network map

1. A sample of the network map is presented below. The size of the circle is related to how often the bacteria or archaea are mentioned across the study.


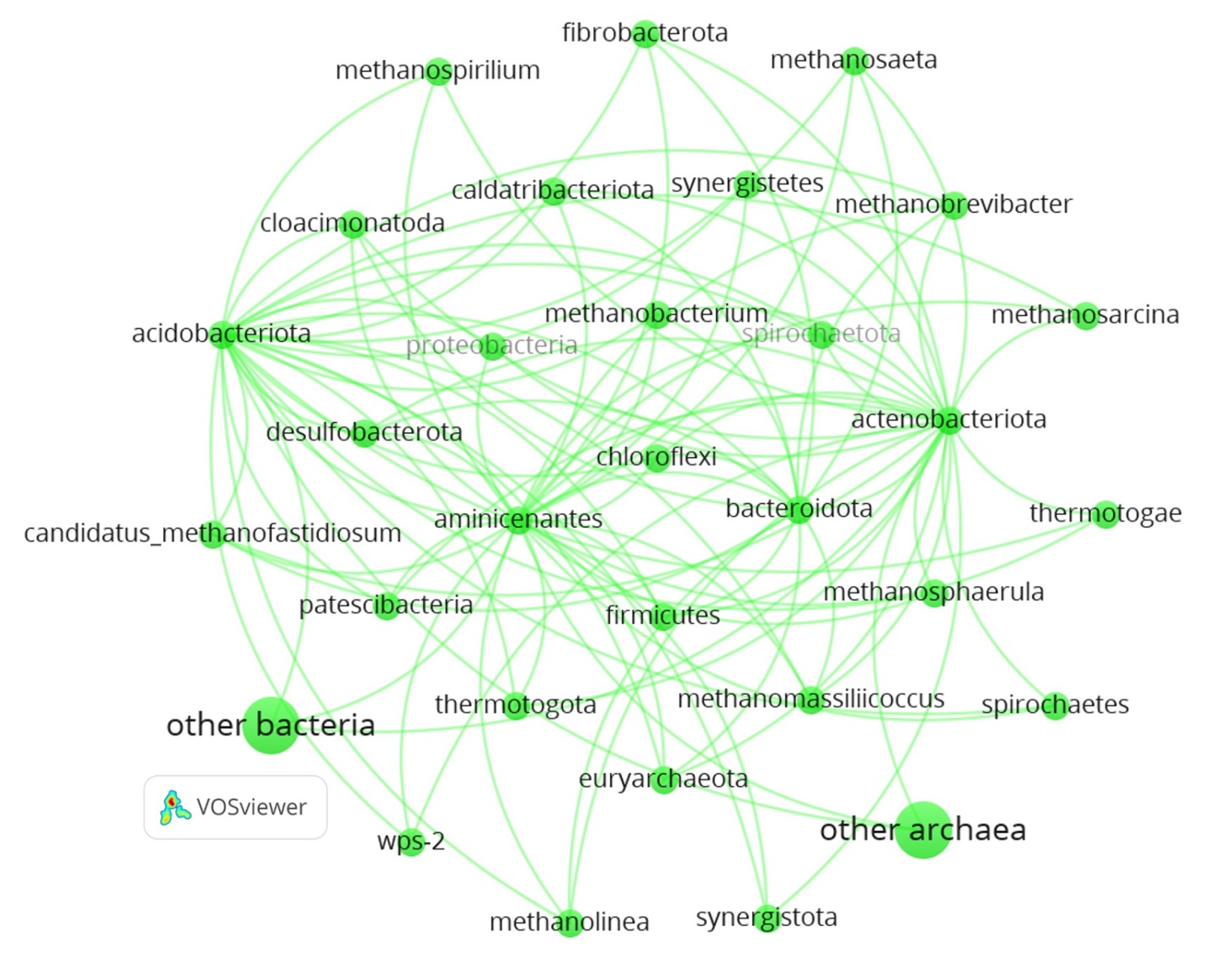


Figure S2. Sample VOSviewer network map of microorganisms.
